# Pneumomediastinum: A Rare Complication of Epidural Analgesia

**DOI:** 10.7759/cureus.18747

**Published:** 2021-10-13

**Authors:** Vibhavari M Naik, Syed Nusrath, Basanth K Rayani, Pratap Reddy, Rohit Yalamnchilli

**Affiliations:** 1 Department of Anaesthesiology, Surgical Critical Care, Pain and Palliative Medicine, Basavatarakam Indo-American Cancer Hospital and Research Institute, Hyderabad, IND; 2 Department of Surgical Oncology, Basavatarakam Indo-American Cancer Hospital and Research Institute, Hyderabad, IND; 3 Department of Radiodiagnosis, Basavatarakam Indo-American Cancer Hospital and Research Institute, Hyderabad, IND

**Keywords:** pneumomediastinum, pneumorrhachis, epidural block, loss of resistance technique, complication, post-operative

## Abstract

Pneumomediastinum is a rare complication following epidural block using the loss of resistance (LOR) technique with air. It is speculated to result from the opening of potential space connecting the epidural space and the posterior mediastinum via intervertebral foramina through fascial planes. To date, only two cases of pneumomediastinum after epidural block have been reported. An incidental finding of pneumomediastinum two days after the procedure has not been reported before. Epidural block as a cause should be considered among multiple causes while interpreting the imaging of this life-threatening complication in the postoperative period.

## Introduction

Epidural analgesia is commonly used for major abdominal surgeries. Loss of resistance (LOR) is an established technique to identify the needle position in the epidural space [1]. The LOR technique entails drawing air or saline into a syringe and applying an intermittent or constant pressure as the Tuohy needle is inserted into the epidural space. As the needle negotiates its way through the ligamentum flavum into the epidural space, a distinct loss of resistance is encountered, and air or saline enters the epidural space confirming the correct position of the needle tip. LOR techniques with air as well as saline are comparable in terms of success rate and complications [2]. The LOR technique with air is still preferred by some anesthesiologists as it provides a better feel of compressibility as well as differentiation from cerebrospinal fluid in case of inadvertent dural puncture [3]. However, using air for LOR has been associated with pneumorrhachis, pneumocephalus, subcutaneous emphysema, venous air embolism, paresthesia, and other neurological complications [4]. We report a case of incidental pneumomediastinum as a rare complication of epidural analgesia. Pneumomediastinum can occur spontaneously due to alveolar rupture or it can be iatrogenic. It can be self-resolving or associated with serious morbidity and mortality [5]. Its presence in the postoperative period should prompt the clinician to look into the possible causes and rule out the life-threatening ones. To date, there have been only two cases of pneumomediastinum attributed to epidural block [6,7]. An incidental finding of pneumomediastinum two days after epidural analgesia has never been reported. With the increasing interest in regional anesthesia and increased usage of epidurals, clinicians must be aware of this entity.

We obtained ethical approval from the Institutional Ethics Committee at the Basavatarakam Indo-American Cancer Hospital and Research Institute and written informed consent from the patient to publish this article. Care has been taken to avoid any patient-identifying information in both text and image.

## Case presentation

A 57-year-old male with no comorbidities and addictions was diagnosed to have carcinoma stomach with gastric outlet obstruction. His body mass index was 22 kg/m^2^ and he was classified as American Society of Anesthesiologists physical status grade II. All his laboratory parameters were normal. His cardiac and pulmonary status was also normal. He underwent laparoscopic-assisted radical distal gastrectomy with perigastric lymph nodal dissection under general anesthesia supplemented with epidural analgesia. Epidural analgesia was administered aseptically in a sitting position at the T 9-10 intervertebral space using a midline approach. An 18-gauge Tuohy needle attached to a syringe filled with about 5 ml of air with intermittent pressure was applied to locate the epidural space using the LOR technique with air. Epidural space was located in the second similar attempt after the redirection of the needle. After confirming the space, the epidural catheter was threaded in. Epidural analgesia with 0.2% ropivacaine at a dose of 8-10 mL/hr was administered throughout the surgery and continued in the postoperative period. The procedure was uneventful.

**Figure 1:**
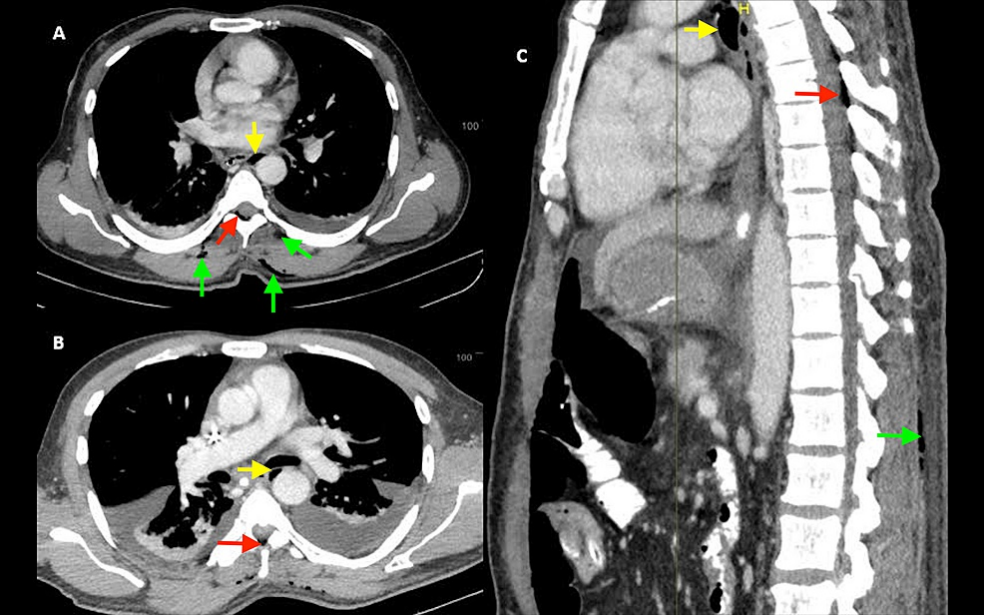
CT scan of the chest The scan images in axial (A, B panels) and sagittal (C panel) planes depicting air in the epidural space (red arrows), subcutaneous tissue intermuscular plane (green arrows), and posterior mediastinum (yellow arrows) CT: computed tomography

On postoperative day two, the patient presented with severe pain (7/10 on the visual analog scale) in the epigastric region with tenderness and abdominal distension, despite continuous epidural analgesia. He was hemodynamically stable. A contrast-enhanced CT of the chest and abdomen was performed to rule out possible anastomotic leak or pancreatitis. Imaging was suggestive of ileus with minimal bilateral pleural effusions and passive basal atelectasis. Incidentally, few air foci were detected in the intermuscular plane in the paravertebral muscles, subcutaneous space, epidural space (T 4-T 10), and the paraaortic and the paratracheal area in the posterior mediastinum (Figure 1). There was no chest pain, dyspnea, desaturation, fever, back pain, or neurological symptoms. There was no leak, disconnection, or accidental air injection in the epidural catheter since its placement. The epidural catheter was then removed, and the patient was closely monitored for neurological complications. He opted for conservative management (antacids, proton pump inhibitor, prokinetic), and was discharged on day 12. A repeat CT scan of the chest and abdomen on day 19 showed complete resolution of epidural and extra-epidural air including the pneumomediastinum.

## Discussion

Pneumorrhachis refers to the presence of intra- or extra-dural air within the spinal canal. Iatrogenic pneumorrhachis may occur with the LOR technique using air during epidural analgesia. The epidural space is a potential space that can communicate with the intervertebral foramina, retroperitoneum, and posterior mediastinum. Due to the negative pressure, the air can get sucked into the epidural space during the procedure. If the volume or the force of air injection during the procedure is high, it may result in air movement into these potential locations. Pneumorrhachis has been demonstrated following epidural analgesia to cause discomfort, pain, neurological deficits, and even cardiac arrest [8-10].

Pneumomediastinum is defined as the presence of extraluminal air in the mediastinum that is generally benign but can become life-threatening in rare cases with progressive accumulation of air [5]. The air can enter the mediastinum from the airway, lungs, pleura, esophagus, peritoneum, and, in rare cases, epidural space. The possible causes of pneumomediastinum in our case could be as follows: air leak due to airway injury, esophageal rupture during preoperative endoscopy, mediastinitis due to a gas-producing organism, or diffusion of peritoneal gas into mediastinum via hiatus, retroperitoneum, or pleural breach during laparoscopic surgery. There was no obvious airway injury and, moreover, an air leak is usually associated with pneumothorax, which was absent in this case. Esophageal rupture during endoscopy was ruled out as pneumomediastinum was not noted on the preoperative CT chest done two days prior to surgery. The patient also did not have any signs of sepsis, and hence the mediastinitis as a cause was ruled out. The pleura and hiatus were not breached during the surgery. The dissection of perigastric nodes including the coeliac nodes during laparoscopic surgery can theoretically cause pneumomediastinum. But, the absence of air in the retrocardiac location and the presence of air in the paraaortic, parabronchial, and paraspinal muscles along with pneumorrhachis, suggests that the pneumomediastinum was likely due to epidural space location. In this case, pneumorrhachis and pneumomediastinum were incidentally detected on CT imaging on postoperative day two. Previous reports have indicated symptoms of pneumorrhachis manifesting immediately or within a few hours [6-10]. Our patient was an asymptomatic case of pneumorrhachis and pneumomediastinum detected two days following surgery. Our case also highlights the fact that air in these potential spaces may persist beyond two days after the procedure. This should be kept in mind while interpreting imaging in the postoperative period. Table 1 illustrates the context, imaging findings, and the outcomes of our case in comparison to the other two cases reported in the literature.

**Table 1:** Pneumomediastinum due to epidural block: a review of the literature HCC: hepatocellular carcinoma; PET: positron emission tomography; TACE: transarterial chemoembolization; PM: pneumomediastinum; T: thoracic vertebra; C: cervical vertebra; PR: pneumorrhachis; PT: pneumothorax

Author, year	Context of epidural analgesia	Symptoms	Imaging findings	Management and outcome
Lim et al., 2013 [6]	56-year-old man with HCC for pain control	Asymptomatic	PET scan performed three hours later for TACE revealed PM from C-2 to T-10 with PR, paravertebral muscle emphysema	Conservative; recovered
Shaik et al., 2021 [7]	26-year-old primigravida for cesarean section	Tachypnea and fall of saturation in the immediate postoperative period	CT chest showed right apical PT and PM, PR in the thoracic region	Conservative; recovered
Our case	57-year-old man for distal gastrectomy	Asymptomatic for PR and PM	CT done for ileus on postoperative day two revealed PR in thoracic region, emphysema in back muscles, and PM	Conservative; recovered

## Conclusions

In rare cases, pneumorrhachis and pneumomediastinum can result from an epidural block using the LOR technique with air. This should be taken into consideration while analyzing the causes of pneumomediastinum in the early postoperative period. During the epidural block, it is prudent to use the least quantity and force of the air. Alternatively, LOR with saline can prevent this complication.
